# Application value of nomogram and prognostic factors of gastric cancer patients who underwent D2 radical lymphadenectomy

**DOI:** 10.1186/s12876-019-1098-6

**Published:** 2019-11-15

**Authors:** Guang-Chuan Mu, Yuan Huang, Zhi-Ming Liu, Xiang-Hua Wu, Xin-Gan Qin, Zhi-Bai Chen

**Affiliations:** 1 Department of Gastrointestinal Surgery, the First Affiliated Hospital of Guangxi Medical University, NO.6 of Shuangyong Road, Qingxiu District, Nanning, 530021 China; 2 Department of General Surgery, the Second Affiliated Hospital of Guangxi Medical University, Nanning, 530007 China

**Keywords:** Gastric cancer, Surgery, Prognosis, Nomogram

## Abstract

**Background:**

The aim of this study was to explore the prognostic factors and establish a nomogram to predict the long-term survival of gastric cancer patients.

**Methods:**

The clinicopathological data of 421 gastric cancer patients, who were treated with radical D2 lymphadenectomy by the same surgical team between January 2009 and March 2017, were collected. The analysis of long-term survival was performed using Cox regression analysis. Based on the multivariate analysis results, a prognostic nomogram was formulated to predict the 5-year survival rate probability.

**Results:**

In the present study, the total overall 3-year and 5-year survival rates were 58.7 and 45.8%, respectively. The results of the univariate Cox regression analysis revealed that tumor staging, tumor location, Borrmann type, the number of lymph nodes dissected, the number of lymph node metastases, positive lymph nodes ratio, lymphocyte count, serum albumin, CEA, CA153, CA199, BMI, tumor size, nerve invasion, and vascular invasion were prognostic factors for gastric cancer (all, *P* < 0.05). However, merely tumor staging, tumor location, positive lymph node ratio, CA199, BMI, tumor size, nerve invasion, and vascular invasion were independent risk factors, based on the results of the multivariate Cox regression analysis (all, *P* < 0.05). The nomogram based on eight independent prognostic factors revealed a well-degree of differentiation with a concordance index of 0.76 (95% CI: 0.72–0.79, *P <* 0.001), which was better than the AJCC-7 staging system (concordance index = 0.68).

**Conclusion:**

The present study established a nomogram based on eight independent prognostic factors to predict long-term survival in gastric cancer patients. The nomogram would be beneficial for more accurately predicting the prognosis of gastric cancer, and provide important basis for making individualized treatment plans following surgery.

## Background

Globally, the incidence of gastric cancer ranks fifth among malignant tumors, and the mortality rate ranks second. Merely in 2015, it was estimated that there were 679,000 new cases of gastric cancer in China, and approximately 498,000 patients with gastric cancer died [1]. D2 radical resection for gastric cancer has attained a global consensus as a standard procedure. At present, the prognosis of patients with gastric cancer after D2 radical resection is mainly evaluated according to the TNM staging system in the 7th and 8th editions of the American Joint Committee on Cancer (AJCC) [2, 3]. However, the AJCC staging system is confined to three indicators: depth of tumor infiltration, lymph node metastasis, and distant metastasis. The influence of age, location and size of tumors, nerve and vascular invasion, physical condition, and serology (serum albumin and tumor markers) on prognosis is neglected, limiting the application of the TNM staging system in clinical practice. Therefore, it is necessary to explore a more comprehensive and accurate evaluation system to predict the prognosis of gastric cancer patients after the operation.

In recent years, nomograms have gained increased attention as strong prognostic statistical models with user-friendly interfaces. The nomogram is a predictive tool, which creates a simple graphical representation of a statistical predictive model that generates a numerical probability of a clinical event. Previous studies [4–6] have reported the advantage of nomograms in predicting the prognosis of tumors, because it is easy to operate, its graphical image is intuitive, and it is easy for clinicians to predict an individualized prognosis.

At present, few studies in China have explored the application value of nomograms in predicting the prognosis of patients with gastric cancer after D2 radical resection. In the present study, the clinical data of 421 patients with gastric cancer, who underwent standard D2 radical resection performed by the same surgical team in our department from January 2009 to May 2017, were retrospectively analyzed, in order to explore the risk factors that affect the prognosis of gastric cancer, and establish a new nomogram model and proofreading curve.

## Methods

### General data

A total of 421 patients with gastric cancer, who underwent standard D2 radical resection performed by the same surgical team in our department from January 2009 to May 2017, were enrolled into the present study as study subjects. All these patients met the following inclusion criteria: (1) patients with gastric cancer confirmed by preoperative gastroscopic biopsy, and with indications for D2 radical resection by preoperative comprehensive evaluation; (2) patients treated with D2 radical resection; (3) patients with complete clinical and pathological data; (4) patients without neoadjuvant therapy; (5) patients with other malignant tumors were excluded. The present study was approved by the Ethics Committee of our hospital (Batch number: 2018 [KY-E-062]).

### Data acquisition

In the present study, the clinical and pathological data of patients were collected and recorded after the operation, The collected data mainly included gender, age, tumor stage, tumor location, tumor size, pathological type, vascular invasion, nerve invasion, Borrmann classification, the number of lymph node metastases, the total number of lymph nodes dissected, positive lymph node proportion, preoperative routine blood test (hemoglobin [Hb], platelet count [PLT], neutrophil count [NEU], lymphocyte count [LYM], neutrophil-lymphocyte ratio [NLR]), serum albumin (Alb), preoperative tumor markers (alpha-fetal protein [AFP], carcinoembryonic antigen [CEA], CA12–5, CA15–3, CA19–9), body mass index (BMI), and celiac artery variations.

### Follow-ups

These patients were followed up for 12.0–112.0 months, with an average duration of 45.6 months. The deadline for the follow-up was May 31, 2018. The follow-up methods included outpatient review and telephone follow-up. In postoperative years 1–2, these patients were followed-up every 3 months, including computed tomography (CT), gastroscopy, tumor markers, and so on. In postoperative years 3–5, these patients were followed-up every six months. Thereafter, these patients were followed-up every 12 months. Postoperative survival time was the time from operation to death, or the deadline for follow-up.

### Statistical analysis

Data were statistically analyzed using statistical software SPSS 16.0. Count data were compared using Chi-square test. Two independent samples *t*-test or univariate analysis of variance were used for the evaluation of normally distributed measurement data. The Kaplan-Meier method was used for calculating the survival rate. The possible factors that can affect the prognosis of gastric cancer were analyzed using Cox hazard regression model univariate analysis. Factors that had statistical significance in the univariate analysis were further analyzed using Cox’s multivariate regression analysis, in order to determine the independent prognostic factors.

The independent risk factors that affected the prognosis of gastric cancer after D2 radical resection were used for the construction of the nomogram prediction model on the “rms” package of the R3.4.0 software. Then, the score of each index was obtained. The corresponding scores of each of the patient’s indexes were added up to obtain the total scores. With the downward matching of total scores, the corresponding 5-year overall survival probability could be obtained. The bootstrap method was used to sample 500 times, and the internal validation of the nomogram model was carried out. The resolution test was carried out by calculating the concordance index (c-index). The C-index estimates the probability of concordance between predicted and observed outcomes in rank order and is equivalent to the area under the receiver–operator characteristic curve (assuming there are no censored cases). The c-index values within 0.7–0.8 indicated a good resolution, while values > 0.8 indicated an excellent resolution. Furthermore, the c-index was compared with that of the gastric cancer staging, according to the 7th edition of the AJCC. The higher the c-index was, the higher the accuracy of the model became. Concordance tests were carried out by drawing the calibration chart of predicted values and actual values through the model. *P* < 0.05 was considered statistically significant.

## Results

### Related factors that affect the prognosis of gastric cancer after D2 radical resection

**Fig. 1:**
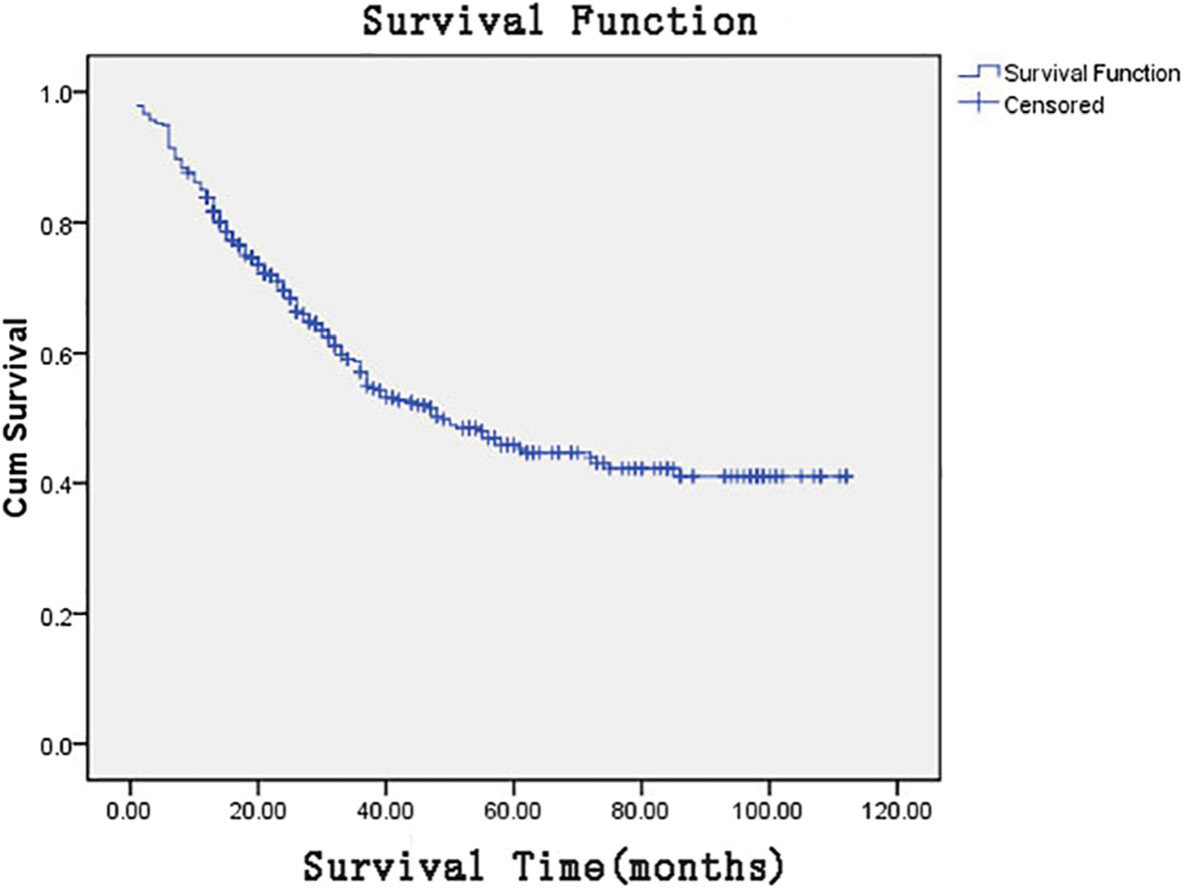
Overall survival curve for all patients

Among these 421 patients, 306 patients (72.7%) were male and 115 patients (27.3%) were female, and the median age of these patients was 56.1 years old (range: 19–86 years old). The 3-year overall survival rate was 58.7%, the 5-year overall survival rate was 45.8%, and the median survival period was 49.00 months (95% CI: 37.56–60.44). The overall survival curve for all patients is presented in Fig. 1. The Cox’s univariate analysis revealed that comprehensive tumor staging, location, size, nerve invasion, vascular invasion, Borrmann classification, the number of lymph node metastases, the total number of lymph nodes dissected, positive lymph node proportion, lymphocyte count, serum albumin, CEA, CA15–3, CA19–9 and BMI were the influencing factors for the prognosis of gastric cancer after D2 radical resection (*P* < 0.05, for all). Furthermore, the comprehensive tumor staging, the location, positive lymph node proportion, CA19–9, BMI, tumor size, nerve invasion and vascular invasion were the independent prognostic factors for gastric cancer after D2 radical resection (P < 0.05, for all; Tables 1 and 2).

**Table 1:** Univariate analysis of the prognosis of gastric cancer after D2 radical resection for 421 cases

Factors	Subgroups	n(%)	Univariate analysis		
			HR	95%CI	*p*
Age	–	421 (100)	1.227	.999–1.505	.051
Gender	Male	306 (72.7)	1		
	Female	115 (27.3)	1.110	.812–1.517	.512
Staging	I	90 (21.4)	1		
	II	109 (25.9)	2.846	1.484–5.458	.002
	III	222 (52.7)	7.618	4.217–13.763	.000
Tumor site	Proximal	51 (12.1)	1		
	Gastric body	59 (14.0)	.664	.408–1.081	.099
	Antrum	281 (66.7)	.444	.305–0.645	.000
	Total stomach	30 (7.1)	.857	.493–1.492	.587
Pathological type	Adenocarcioma	369 (87.6)	1		
	Mucous carcinoma	15 (3.6)	.919	.431–1.958	.827
	Gastric signet ring cell carcinoma	15 (3.6)	1.320	.675–2.581	.418
	Undifferentiated tumor	22 (5.2)	.973	.528–1.791	.929
Tumor size (cm)	–	–	1.124	1.079–1.170	.000
Neural invasion	No	284 (67.5)	1		
	Yes	137 (32.5)	2.535	1.901–3.382	.000
Vessel invasion	No	271 (64.4)	1		
	Yes	150 (35.6)	2.909	2.187–3.870	.000
Borrmann type	I	19 (4.5)	1		
	II	105 (24.9)	1.550	.464–5.182	.476
	III	218 (51.8)	4.995	1.588–15.717	.006
	IV	79 (18.8)	6.106	1.900–19.620	.002
Number of lymph nodes metastasis	–	421 (100)	1.074	1.058–1.090	.000
Total lymph nodes	–	421 (100)	1.018	1.004–1.032	.014
The rate of positive lymph nodes	–	421 (100)	12.072	7.514–19.393	.000
BMI(kg/m^2^)	< 18.5	66 (15.7)	1		
	18.5–24.0	283 (67.2)	.560	.396–0.793	.001
	≥24.0	72 (17.1)	.393	.241–0.640	.000
Variation of coeliac artery	No	311 (73.9)	1		
	Yes	110 (26.1)	1.039	.755–1.430	.815
Hb(g/L)	< 130	295 (70.1)	1		
	≥130	126 (29.9)	.803	.585–1.104	.177
PLT(10^9^/L)	< 125	16 (3.8)	1		
	125–350	335 (79.6)	.736	.376–1.442	.372
	> 350	70 (16.6)	.892	.428–1.861	.761
NEU(10^9^/L)	< 1.8	17 (4.0)	1		
	1.8–6.3	361 (85.7)	.590	.320–1.088	.091
	> 6.3	43 (10.2)	.701	.341–1.441	.334
LYM(10^9^/L)	< 1.1	44 (10.5)	1		
	1.1–3.2	373 (88.6)	.606	.403–0.910	.016
	> 3.2	4 (1.0)	.893	.121–6.597	.912
NLR	< 2.37	252 (59.9)	1		
	≥2.37	169 (40.1)	1.153	.869–1.531	.324
Alb(g/L)	< 35	121 (28.7)	1		
	≥35	300 (71.3)	.587	.440–0.783	.000
AFP(μg/L)	< 20	407 (96.7)	1		
	≥20	14 (3.3)	1.331	.656–2.703	.428
CEA(μg/L)	< 5	341 (81.0)	1		
	≥5	80 (19.0)	1.986	1.446–2.726	.000
CA125(kU/L)	< 35	396 (94.1)	1		
	≥35	25 (5.9)	1.555	.885–2.731	.125
CA153(kU/L)	< 31.3	412 (97.9)	1		
	≥31.3	9 (2.1)	2.789	1.372–5.669	.005
CA199(kU/L)	< 37	352 (83.6)	1		
	≥37	69 (16.4)	2.479	1.786–3.442	.000

**Table 2:** Multivariate analysis of the prognosis of gastric cancer after D2 radical resection

Variables	Subgroups	B	SE	Wald	HR	95%CI	*P* Value
Staging	I			6.294	1	–	.029
	II	0.636	.338	3.530	1.889	.973–3.667	.060
	III	0.866	.346	6.279	2.379	1.208–4.684	.012
Tumor site	Proximal			9.359	1	–	.038
	Gastric body	−0.596	.254	5.482	.551	.335–.908	.019
	Antrum	−0.532	.199	7.153	.588	.398–.868	.007
	Total stomach	−0.665	.291	5.236	.514	.291–.909	.022
Tumor size	–	0.060	.029	4.168	1.061	1.002–1.124	.041
Neural invasion	–	0.342	.168	4.159	1.408	1.013–1.957	.041
Vessel invasion	–	0.631	.162	15.146	1.879	1.368	.000
The rate of positive lymph nodes	–	1.195	.348	11.791	3.302	1.670–6.531	.001
BMI(kg/m^2^)	< 18.5	–	–	9.370	1	–	.013
	18.5–24.0	−0.498	.185	7.258	.608	.423–.873	.007
	≥24.0	−0.695	.258	7.253	.499	.301–.828	.007
CA199(kU/L)	–	0.591	.173	11.595	1.805	1.285–2.536	.001

### Construction and evaluation of the nomogram prediction model

**Fig. 2:**
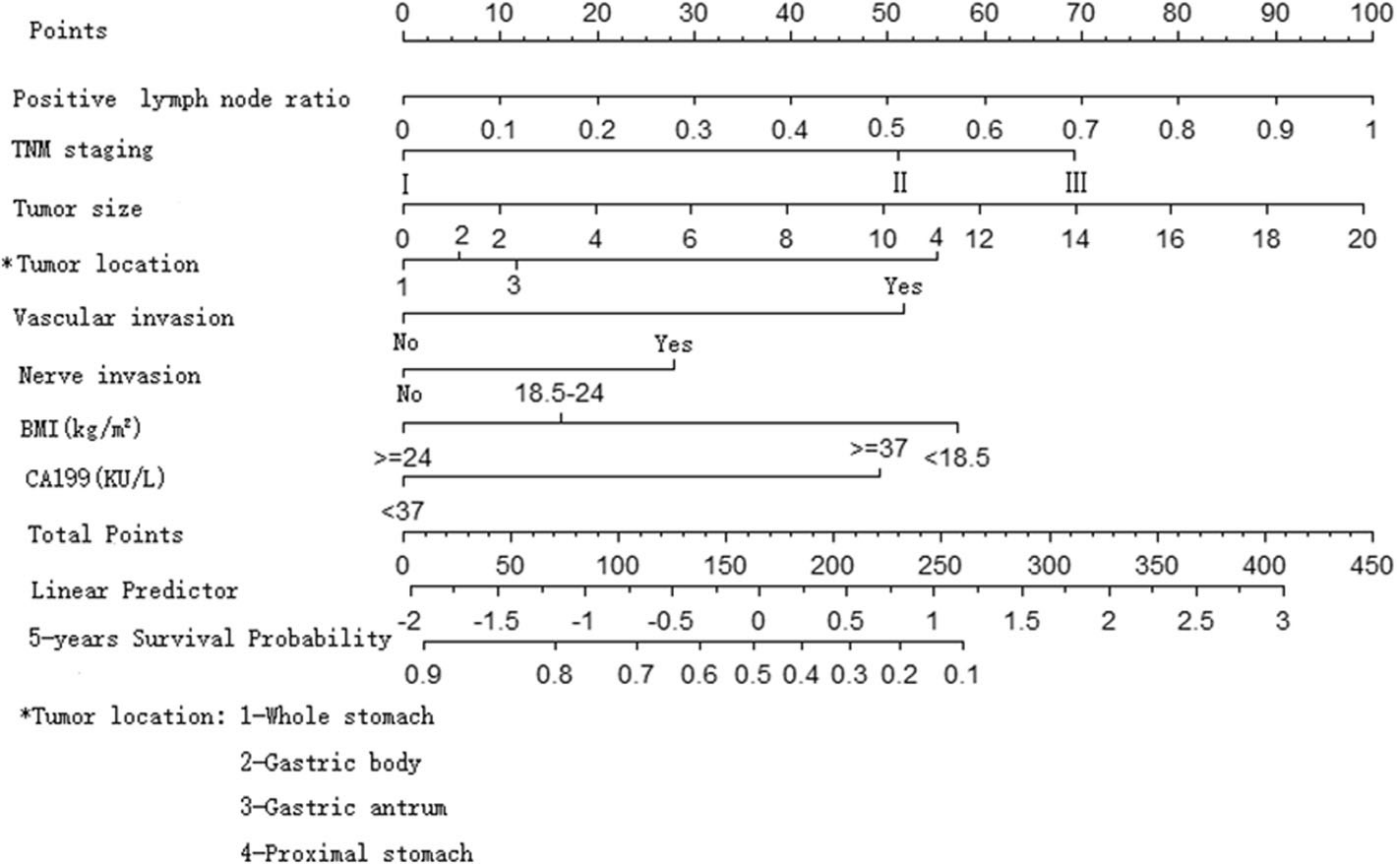
The nomogram model for predicting the prognosis of patients with gastric cancer after d2 radical resection

**Fig. 3:**
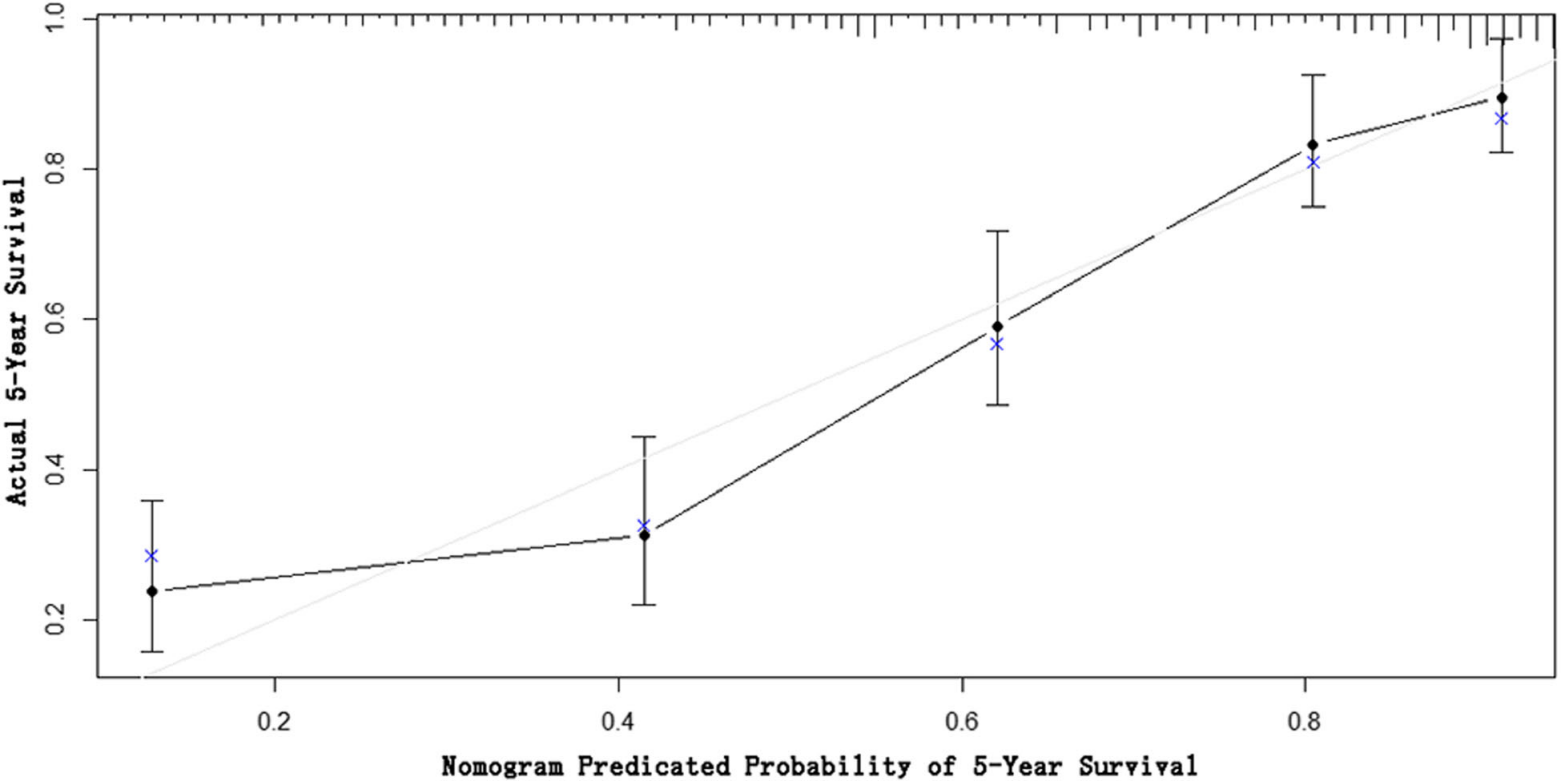
The calibration charts for comparing the 5-year survival rate predicted by the nomogram with the actual 5-year survival rate

The R software was used to construct the nomogram model based on the Cox multivariate analysis results to predict the prognosis of patients with gastric cancer after D2 radical resection (Fig. 2). According to the nomogram, stage I scored 0 point, stage II scored 50.5 points, and stage III scored 69 points. The tumors in the whole stomach scored 0 point, the tumors in the gastric body scored 6 points, the tumors in the gastric antrum scored 11 points, and tumors in the proximal end scored 55 points. Furthermore, vascular invasion scored 51.5 points and nerve invasion scored 28.5 points. In addition, BMI < 18.5 scored 57.5 points, BMI within 18.5–24.0 scored 16 points, and BMI > 24.0 scored 0 points. Moreover, positive CA19–9 scored 49 points. With the increase in the proportion of positive lymph nodes and the largest diameter of tumors, the corresponding score of the prediction model increased. The total score of each patient matched downward to the “the 5-year survival rate” survival axis. Thus, the 5-year survival rate could be predicted. The internal verification revealed that the c-index of the model was 0.76 (95% CI: 0.72–0.79, *P* < 0.001). This suggests that the model was well-established, and the c-index was higher than that of the 7th edition of AJCC staging (0.68). The concordance tests were carried out by drawing the calibration chart of predicted values and actual values. The results suggest that the 5-year survival rate predicted by the nomogram model was well-correlated to the actual 5-year survival rate (Fig. 3).

## Discussion

The TNM staging system of AJCC is the most widely used staging system at home and abroad at present. Furthermore, it plays an important role in the prognosis assessment and decision-making of follow-up treatment after the operation, and it has been constantly updated and improved to the present 8th Edition [3]. The TNM staging system based on anatomy was confined to three indicators: primary lesion, lymph node metastasis, and distant metastasis. Adjustments in the new edition also failed to significantly improve the ability to predict the prognosis of gastric cancer. Therefore, it is necessary to explore a more comprehensive and accurate prediction and evaluation system to individually predict the prognosis of gastric cancer patients in clinic [7, 8]. Studies have put forward that [9, 10] the TNM staging system combined with tumor molecular biology and genetics can significantly improve the ability to predict the prognosis of gastric cancer. However, it is often complicated and has high economic cost, limiting its clinical application. Except for TNM, the post-operative long-term survival rate of gastric cancer is also influenced by many factors, and a prediction model combined with other factors may better predict the prognosis of gastric cancer patients.

In the present study, the potential factors that influence the prognosis of gastric cancer after the operation, such as age, nutritional status, routine blood test results, biochemical indicators, tumor markers, pathology and tumor anatomy, were collected to seek for risk factors that affect the prognosis of gastric cancer after D2 radical resection. The multivariate Cox’s proportional hazard regression analysis revealed that age, gender, tumor pathological type, the number of lymph node metastasis, the total number of lymph nodes dissected, hemoglobin, blood cell count, neutrophil-lymphocyte ratio and serum albumin were not correlated to the prognosis of patients with gastric cancer after D2 radical resection. In addition to the TNM-determined comprehensive tumor staging, which is a recognized independent prognostic factor, the present study revealed that the larger the proportion of positive lymph nodes and the largest the diameter of tumors, the higher the risk of death in gastric cancer patients after D2 radical resection. Hence, the proportion of positive lymph nodes and size of tumors can be used as supplements to the TNM staging system, which can help better predict the prognosis of patients with gastric cancer after the operation [11, 12]. In the present study, tumor location was also an independent risk factor for the prognosis of gastric cancer after D2 radical resection. The risk was highest when the tumors were located in the proximal end, followed by those located in the gastric antrum, gastric body and whole stomach. However, the risk was higher when the tumors were located at the gastric antrum, when compared to tumors located at the gastric body and whole stomach. This is different from the conclusions of previous studies [13, 14]. The reason may be the small sample size of the present study.

Nerve and vascular invasion is an important pathological parameter in patients with gastric cancer, which has important clinical value in judging the prognosis of patients and guiding treatment. The results of the present study revealed that nerve and vascular invasion was also an independent risk factor for the prognosis of gastric cancer after D2 radical resection, and the risk of vascular invasion was higher than that of nerve invasion (the nomogram revealed a result of 51.5 vs. 28.5). This is consistent with the results of a previous study. The results of the meta-analysis, which involved 30,590 patients with gastric cancer after surgery, revealed that the incidence of nerve invasion was as high as 40.9%, and was an independent risk factor for the overall survival rate and disease-free survival rate of gastric cancer after surgery, and that nerve invasion was mainly affected by lymph node metastasis, tumor size, depth of the tumor invasion, and other pathological factors [15]. Luchuan Chen et al. [16] analyzed the prognosis of 1801 patients with gastric cancer after the operation, and the results revealed that the 5-year survival rate of patients with vascular or nerve invasion was significantly lower than that of patients without vascular and nerve invasion, and that the 5-year survival rate decreased more significantly in patients with positive vascular and nerve invasion. Hwang JE et al. similarly considered that [17] the coexistence of vascular and nerve invasion was an independent factor that influenced the survival rate of patients with stage II and III gastric cancer after D2 radical resection. Hence, the combination of these two could more accurately predict the prognosis of patients with gastric cancer, and could be used as one of the indications of adjuvant therapy after the operation.

The effect of preoperative serum tumor markers on the prognosis of patients with gastric cancer after the operation remains controversial. A previous study revealed that CEA and CA19–9 were the most commonly used indicators for the early diagnosis and monitoring of recurrence of gastric cancer after an operation [18]. The results of the study conducted by Lin JX et al. revealed that [19] preoperative CEA combined with CA19–9 level could be used as an independent predictor of prognosis for patients with resectable gastric cancer, and the modified TNM staging system with preoperative CEA/CA19–9 levels could more accurately predict the prognosis of patients with stage III gastric cancer after D2 radical resection. In the present study, the univariate analysis revealed that CEA, CA19–9 and CA15–3 were the influencing factors for the prognosis of gastric cancer after the operation. However, the multivariate analysis revealed that only elevated CA19–9 was an independent risk factor for the prognosis of gastric cancer after D2 radical resection. CA19–9 could be used as an index to predict the prognosis of patients with gastric cancer after the operation, and monitor the recurrence after the operation.

The limitations of this study include its retrospective nature, the small sample size of non-adenocarcinoma tumors, the lack of data on subgroup analysis, and the fact that negative margins was not assessed as a data point. Due to these limitations, our outcomes may not be clinically applicable in all situations.

## Conclusion

In the present study, the 5-year survival rate of patients with gastric cancer was predicted well by the prognostic nomogram model of patients with gastric cancer after D2 radical resection. This was established based on comprehensive staging, tumor location, tumor size, nerve invasion, vascular invasion, positive lymph node proportion, BMI and CA19–9, and the c-index was 0.76. Furthermore, the predictive capability was better than that of the TNM staging system of AJCC, which could predict individually for patients, and be helpful in formulating individualized treatment decision-making for patients in clinic. However, the present study was a single-center retrospective study. Hence, the conclusion needs an external cohort study or a multi-center prospective study to further validate its clinical application value.

## Data Availability

The datasets used and/or analysed during the current study available from the corresponding author on reasonable request.
